# The Process of Digestion

**Published:** 1891-05

**Authors:** 


### THE PROCESS OF DIGESTION.

The grinding process of food, so essential to its fitness to enter the stomach, is carried on by the teeth.

Saliva is an aid to this breaking up. It moistens the food and makes a partial solution of the particles. If it were not for its presence, the mouth would be dry, and it would be very difficult to masticate the food.

There is a continuous canal running completely through the body, which is more winding in its course than the most crooked stream. It is called the alimentary (food) canal.

The solid food, broken up by the grinding mill, passes through the alimentary canal in a liquid form. This canal is about 28 feet long and has many subdivisions.

These are the mouth, the pharynx, œsophagus, stomach, duodenum, jejunmum, ileum, cæcum, colon and rectum.

The alimentary canal is lined throughout with mucous membrane, which is full of blood vessels and secreting glands.

The secretions from these glands dissolve the food, and the blood vessels, which are tributaries to the alimentary canal, absorb the nourishment from the food solution as fast as it is formed, and convey it to the tissues of the different organs of the body.

In the walls of this canal is a muscular layer which causes the canal to contract around the particles of food, thus forcing it gradually along its whole length.

Opening into the mouth are the openings of three sets of salivary glands, the parotid, the sublingual and the submaxillary.

The parotid glands have openings on the inner side of the cheeks, the sublingual under the tongue, and the submaxillary around the jaws.

When the mouth is at rest, the salivary glands secrete a small but constant amount of fluid, and when the jaws are in motion the flow is rapid. The total amount of saliva secreted in one day is about two and a half pints. The same is true of the other glands, which secrete more abundantly when there is work for them than when in a state of idleness. After the food has been "chewed" and moistened sufficiently in the mouth, it drops into the pharynx (throat), and is passed along to the stomach by means of the œsophagus. As the food drops into the stomach, a valve closes behind it and shuts it in. At the other end of the stomach is the pyloric valve.

These two valves, one at either end of the stomach, retain the food in that organ, while digestion is going on. When food has been deposited within the stomach the glands in the walls at once secrete the gastric juice.

The muscular coat of the stomach is quite complicated. The contractions of this muscular layer produce a varied motion to the contents of the stomach. The food is caused to pass along the sides of the stomach and back through the centre, thus bringing all particles of food into close action with the gastric juice.

By this provision in the structure of this organ an important function is established. When the stomach is empty it is in a state of collapse, and its walls are in opposition. When filled with food it is distended in the form of a sack or bag about one foot long by six inches across.

The liquid solution of the food as made in the stomach, is called chyme. The inner wall of the stomach, when it is empty, is thrown into rough ridges, but when it is distended the wall is stretched smooth and shows little openings on its surface. These openings are small depressions from 1-100th to 1-200th of an inch in diameter. In the sides and bottom of these depressions are minute orifices, which are the mouths of the gastric glands.

At the upper and larger portion of the stomach is the cardiac valve, and at the smaller and lower end is the pyloric valve.

The upper valve opens to let food enter the stomach, the lower valve opens to let the food pass out of the stomach.

An interesting fact about the pyloric valve is that the side toward the interior of the stomach is covered with mucous membrane similar to that lining the stomach, while the other side is covered with villi, which project from the surface, and are about half a line in length.

The gastric juice has been found to be a colorless liquid, or of an amber hue. Its chief elements are water 975 parts, free acid 4.78 parts, and pepsin 15 parts. Other elements in minute quantities are potassium, lime, ammonium, magnesia and iron.

The reaction of the gastric juice is strongly acid, owing to the presence of free hydrochloride acid, and as some authorities assert, lactic acid, also. The other important element is pepsin.

The pepsin is precipitated from the gastric juice, if some of the bile flows back into the stomach through the pyloric orifice, instead of passing along the intestine, as it should.

Digestion is stopped if either the acid or the pepsin is absent, hence both must be present in solution for the gastric juice to do its work efficiently.

If one has dyspepsia, either there is bile present in the stomach, or the gastric juice has lost its acidity.

The chief characteristic of the gastric juice is that it digests albuminous substances. Examples of this work are the dissolving of the white of an egg, the making liquid of the caseine of cheese, while the oily particles are set free.

The stomach digests certain portions of the food, the small intestine a certain part, and the large intestine carries away the waste materials.

Albumen may be present in many varieties of food ; in meats, grains, fruit and in vegetables. The teeth break up the food, the saliva makes soft paste of the food, and the gastric juice converts the albumen contained in the different kinds of food into albuminose (albumen in solution).

Boiling a vegetable greatly aids the process of digestion.

The gastric juice is acid, the blood alkaline, and as the walls of the stomach are well supplied with blood vessels, the gastric juice cannot react on the stomach, because an alkali and an acid will neutralize each the other.

Now we have the stomach occupied with gastric juice and the albumen of food in solution, also the oily matter and starch, apart by themselves and mingled with the other liquid matter. The pyloric valve opens and all of the contents of the stomach pass into the intestine. The pyloric valve closes and the stomach quiets down.

Opening into the small intestine are two or more ducts or tubes. One of these ducts introduces the bile from the liver into the small intestine.

A little lower down are two openings, the ends of the pancreatic ducts, which permit the flow of the pancreatic juice into the intestine. The important element of the pancreatic juice is the pancreatine. This is capable of digesting the oils and fats, and of changing the starch to sugar.

The bile is present here, and it is supposed that it aids to a great extent as a cathartic in washing out the bowels. The pancreatic juice makes a white, milky emulsion of the fats.

We now have a mixed solution in the small intestine, which is of small calibre and about 20 feet long. On the inner surface are millions of villi, which project into the cavity of the intestine. These villi are traversed by minute blood vessels.

The albuminose, the emulsion of fats and the sugar are absorbed by osmosis directly into the blood in the blood vessels of the villi. Osmosis means the passing of a liquid through a tissue. The gastric juice is also absorbed from the intestine by these villi, and is secreted again into the glands of the stomach as the albumen is taken up from the gastric juice by the blood. Thus we get a circulation of this gastric juice. It is secreted in the stomach, dissolves albumen and passes into the small intestine, is absorbed by the blood vessels and is again taken up by the gastric glands, where it is ready to be secreted in the stomach again when there is a demand for it.

After the albuminose, the fat emulsion and the sugar have been absorbed from the small intestine the debris, in the form of vegetable tissues, undigested animal tissues and the waste from the process of digestion, is passed on to the large intestine.

Food should be properly cooked, and slowly and thoroughly masticated. After a hearty meal the best thing to do is to sit quietly back in an easy chair and doze, while digestion is going on, or for a time at least, until digestion is well under way.

The dinner should come at that part of the day when the most time can be given to it.

Nitrogen and carbon are the two principal ingredients of food. Nitrogen supplies the waste tissues and carbon furnishes heat.

Plants and vegetables inhale carbonic acid gas and exhale oxygen.

Animals inhale oxygen and exhale carbonic acid gas.

Man is no exception to the rule.

